# Radiomics Applied to the Diagnosis of Peripheral Nerve Disorders: A Systematic Review and Meta-Analysis of the Existing Literature

**DOI:** 10.3390/jcm15093262

**Published:** 2026-04-24

**Authors:** Veronica Armato, Maria Elena Susi, Riccardo Picasso, Marta Macciò, Federico Pistoia, Federico Zaottini, Carlo Martinoli, Giulio Ferrero, Bianca Bignotti, Alberto Stefano Tagliafico

**Affiliations:** 1 Complex Structure Radiology Ponente, Santa Corona Hospital, 17027 Pietra Ligure, Italy; veronicaarmato16@gmail.com; 2 (DIMES) Department of Experimental Medicine, University of Genoa, 16132 Genoa, Italy; 3 IRCCS Azienda Ospedaliera Metropolitana, 16132 Genoa, Italy; riccardopicasso@gmail.com (R.P.); marta.maccio@gmail.com (M.M.); federico.pistoia@aomliguria.it (F.P.); federico.zaottini@aomliguria.it (F.Z.); carlo.martinolir@unige.it (C.M.); bianca.bignotti@aomliguria.it (B.B.); albertotagliafico@gmail.com (A.S.T.); 4 (DISSAL) Department of Health Sciences, University of Genoa, 16132 Genoa, Italy; 5 Unit of Diagnostic and Interventional Radiology, Santa Corona Hospital, 17027 Pietra Ligure, Italy; giulio.ferrero@gmail.com

**Keywords:** imaging, radiomics, artificial intelligence, peripheral nerves, ultrasound, magnetic resonance imaging

## Abstract

**Background**: This study aims to systematically review the current literature on the application of radiomic features and artificial intelligence (AI) in the diagnosis and prognosis of common peripheral nerve-related conditions, including carpal tunnel syndrome (CTS), chronic inflammatory demyelinating polyneuropathy (CIDP), polyneuropathy, organomegaly, endocrinopathy, monoclonal gammopathy and skin abnormalities (POEMS) syndrome, and in distinguishing between benign and malignant tumors. **Methods**: A comprehensive literature search was conducted in PubMed and Google Scholar for studies published between January 2019 and September 2025. Inclusion criteria comprised studies that used radiomics or AI-based radiomics approaches with diagnostic or prognostic purposes in peripheral nerve disorders. **Results**: A total of 40 studies were identified, of which 17 met the inclusion criteria. Among these, 9 studies employed magnetic resonance imaging (MRI), including one combined with PET/CT, while 8 used ultrasound (US). Most studies were retrospective and limited by small sample sizes, lack of external validation, and predominance of single-center designs. **Conclusions**: Since a seminal study published in 2019, there has been increasing evidence supporting the role of radiomics and AI in improving the diagnosis and prognosis of peripheral nerve disorders, particularly using MRI and US. However, significant challenges remain, including variability in imaging data, segmentation complexity, and limited availability of validated datasets. Future advancements in imaging technologies and multidisciplinary collaboration are essential to enhance clinical applicability.

## 1. Introduction

Radiomics focuses on the extraction of quantitative features from medical images such as, magnetic resonance imaging (MRI), ultrasound (US), and other diagnostic imaging technologies, to be used in different medical and surgical conditions, in addition to standard human-eye evaluation [1,2,3,4,5]. Radiomics provides insights beyond what is visible to the human eye, thereby enhancing diagnostic, prognostic, and therapeutic personalization opportunities [1,2,3,4,5,6,7,8]. One of the first scientific papers on the subject was published in 2012, titled “Radiomics: extracting more information from medical images using advanced feature analysis”, authored by Philippe Lambin et al. [6]. In this work, the radiomics workflow was outlined, from image acquisition to quantitative feature extraction and predictive model development. In the following years, the topic gained increasing scientific interest. A 2022 article published in Radiology by Peter Steiger [7], titled “Radiomics and Artificial Intelligence: from Academia to Clinical Practice”, highlighted the exponential growth in radiomics research and its progressive translation toward clinical practice, with applications spanning brain, breast, lung, and gastrointestinal imaging. However, it was only in 2019, in a relatively new and promising area of radiomic research, that studies began to emerge on the application of radiomics to the diagnosis and prognosis of peripheral nerve disorders, which had previously been primarily evaluated using nerve conduction studies and conventional imaging [8,9,10,11,12,13,14].

Regarding peripheral nerve disorders, the main conditions studied within radiomics include: carpal tunnel syndrome (CTS), a compressive neuropathy of the median nerve at the wrist; chronic inflammatory demyelinating polyneuropathy (CIDP), an immune-mediated neuropathy characterized by progressive motor and sensory deficits; POEMS syndrome, a rare paraneoplastic disorder defined by polyneuropathy, organomegaly, endocrinopathy, monoclonal gammopathy, and skin changes; benign and malignant peripheral nerve sheath tumors (BPNST/MPNST), neoplasms originating from peripheral nerve elements; and diabetic peripheral neuropathy (dpn) and diabetic tibial neuropathy (dtn), the most common peripheral nerve complications of diabetes. Radiomics applied to these conditions aims to improve early diagnosis, characterize tumor malignancy, grade the severity of neuropathy, and predict postoperative outcomes—tasks in which conventional imaging often reaches its interpretive limits. The present systematic review represents one of the first comprehensive quantitative syntheses of radiomic diagnostic accuracy across peripheral nerve disorders, providing a structured evidence base to guide future research priorities and clinical translation.

Therefore, the objective of this article is to provide a systematic review of the current published research on the application of radiomics in diagnosing and evaluating the most frequent peripheral nerve disorders using commonly available clinical techniques such as MRI and US, to identify their potential, challenges, and future directions.

## 2. Materials and Methods

This systematic review was conducted and reported in accordance with the PRISMA 2020 (Preferred Reporting Items for Systematic Reviews and Meta-Analyses) guidelines [15]. The review protocol was not prospectively registered. 

IRB approval was not required due to the nature of the study. The PRISMA reporting guidelines provide guidance for the reporting of systematic reviews evaluating the effects of interventions. Radiomics-based approaches were considered as the index test in this review. The PRISMA checklist is presented in the Supplemental Material (Table S1).

### 2.1. Searching Strategies

A systematic bibliographic search was conducted using the following major medical databases: PubMed and Google Scholar. The search strategy included the following keywords: “Radiomics” AND “peripheral nerve”. The search was initially performed by one reviewer, verified by a second reviewer after standardized training, and updated through September 2025. It should be noted that the search strategy relied on free-text keywords without the use of Medical Subject Headings (MeSH) terms or Boolean expansion operators, which represents a methodological limitation acknowledged in the Limitations section. No other restrictions were applied in order to be as inclusive as possible.

### 2.2. Inclusion and Exclusion Criteria

Inclusion criteria:

Use of radiomics or artificial intelligence linked to radiomics.

Diagnosis or prognosis.

Diseases strictly related to peripheral nerve disorders.

Exclusion criteria:

Lack of use of radiomics or artificial intelligence linked to radiomics.

Diseases NOT strictly related to peripheral nerves.

### 2.3. Study Selection

Two reviewers, independently, with 1 year and 10 years of experience in medical research (V.A.; G.F.), respectively, examined the article titles and abstracts to select studies based on predefined inclusion criteria. Once the articles meeting all the criteria described above were selected, key study components were analyzed and reported in an Excel spreadsheet [Microsoft. Redmond, WA, USA] to describe relevant study characteristics. Discrepancies and controversies were resolved by the senior author (A.T.), with 14 years of experience in medical research.

### 2.4. Data Extraction

One reviewer (V.A.), after standardized training, extracted the following data from the analyzed articles: first author, publication year, publication data, purpose/objective, method, type of scan or sequence used, segmentation, pathology, final goal, study type, ethical committee approval, informed consent, number of patients, hospital, years of the study, inclusion/exclusion criteria, statistics, radiomic features, platform, area under the curve (AUC), sensitivity/specificity, results, conclusions, and limitations. Data were extracted manually by one reviewer (V.A.) and subsequently verified by the second independent reviewer (G.F.). Discrepancies were resolved by consensus or referral to the senior author (A.T.).

### 2.5. Risk of Bias and Quality Assessment

Risks of bias and quality assessment were evaluated to identify potential study design flaws, assess the validity of findings, or detect a potential overestimation of study results. Indeed, if several studies in a systematic review are assessed as having a high risk of bias, results should be interpreted with caution. Risk-of-bias assessments were visualized using the robvis package in R (version 4.5.3) tool was used, a website that provides various tools to analyze bias. By uploading a predefined Excel file, the tool generates a table displaying results in different Cochrane color schemes. The QUADAS-2 tool was employed. The studies are listed in rows, while the following risks of bias are shown in columns:

D1: Patient selection

D2: Index test (radiomics methodology)

D3: Reference standard

D4: Flow and timing

In the final column, an overall quality score is:

35% of the studies achieved a medium-quality score.

35% of the studies were rated as low quality.

30% of the studies achieved a high-quality score.

### 2.6. Data Synthesis and Statistical Analysis

Study characteristics were summarized using descriptive statistics, with categorical variables presented as frequencies and percentages and continuous variables as mean ± standard deviation or median (interquartile range). Random-effects meta-analysis using the DerSimonian–Laird method calculated pooled AUC estimates with 95% confidence intervals, weighting studies by inverse variance. No differential weighting was applied to studies based on methodological quality; instead, the impact of study quality on pooled estimates was assessed through pre-specified sensitivity analyses restricting the analysis to high- and medium-quality studies *(n* = 11), yielding a pooled AUC of 0.892, virtually identical to the primary analysis (0.888). For studies without reported confidence intervals, a conservative variance of 0.01 was assumed and tested through sensitivity analyses. Between-study heterogeneity was quantified using Cochran’s Q test, I^2^ statistic, and tau-squared. Pre-specified subgroup analyses explored heterogeneity by imaging modality, pathology type, segmentation method, study quality, sample size, and validation strategy, with between-group differences tested using meta-regression. Sensitivity analyses assessed robustness through leave-one-out analysis and restriction to high-quality, externally validated, and large studies. Publication bias was evaluated by funnel plot inspection, Egger’s regression test, and the trim-and-fill method. Analyses were performed using Python version 3.12 with pandas, numpy, scipy, matplotlib, and seaborn libraries following PRISMA 2020 and PRISMA-DTA guidelines. Statistical significance was set at alpha equals 0.05 for two-tailed tests, except heterogeneity and publication bias tests using alpha equals 0.10. We acknowledge that pooling AUC estimates across studies addressing different clinical questions—including entrapment neuropathies, nerve tumor characterization, inflammatory neuropathies, and prognostic tasks—introduces clinical heterogeneity that statistical indices such as I^2^ cannot fully capture. The overall pooled AUC should therefore be interpreted as a global descriptive summary of radiomic diagnostic performance in peripheral nerve imaging rather than a clinically actionable estimate for any specific condition. Subgroup analyses stratified by pathology type are considered the primary clinically interpretable results.

## 3. Results

A total of 870 records were identified (847 from PubMed and 23 from other sources). After the removal of 234 duplicates, 636 records were screened. Of these, 596 were excluded, leaving 40 full-text articles assessed for eligibility. Twenty-three studies were excluded (5 not using radiomics and 18 not focused on peripheral nerve disorders), resulting in seventeen studies included in the systematic review and meta-analysis (Table 1).

### 3.1. Study Identification Results

**Figure 1:**
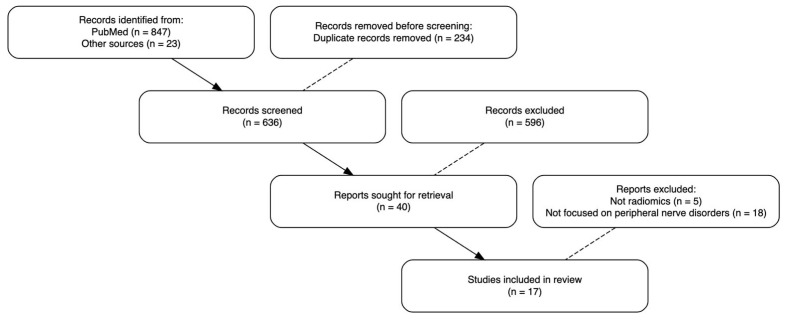
Shows the PRISMA 2020 flow diagram of study selection.

The systematic search identified 847 records from electronic databases and 23 additional records from other sources, yielding 870 records in total. After removing 234 duplicates, 636 unique records were screened based on titles and abstracts. Of these, 596 records were excluded as clearly not meeting eligibility criteria, leaving 40 full-text articles for detailed assessment. Upon full-text review, 23 articles were excluded for the following reasons: five studies did not employ radiomics methodology, and eighteen studies were not focused on peripheral nerve disorders. Finally, seventeen studies met all inclusion criteria and were included in the systematic review and meta-analysis. Inter-rater agreement for study selection was substantial with a Cohen’s kappa coefficient of 0.78. The study selection process is illustrated in the PRISMA flow diagram in Figure 1.

### 3.2. Study Characteristics

The seventeen included studies were published between 2019 and 2025, with a notable temporal trend showing increased publication frequency over time. Two studies were published in 2019, three in 2021, three in 2022, four in 2023, two in 2024, and three through September 2025. This progressive increase reflects a growing research interest in radiomics applications for peripheral nerve imaging. The majority of studies employed retrospective designs, accounting for thirteen studies, 76.5% of the total, while four studies were prospective in design. Regarding study setting, eight studies were single-center investigations without external validation, seven were multi-center studies, and two did not specify this information.

Patient sample sizes varied considerably across studies. Three studies enrolled 100 or fewer patients, eight studies included between 101 and 200 patients, and six studies exceeded 200 patients. Where reported, the total patient population across all studies exceeded 2800 individuals. The pathological conditions investigated were diverse, with carpal tunnel syndrome being the most frequently studied condition, comprising six studies, 35.3% of the total. Peripheral nerve tumors, including differentiation between benign and malignant lesions, were examined in five studies, representing 29.4% of the literature. Four studies focused on various neuropathies, including diabetic peripheral neuropathy, diabetic tibial nerve neuropathy, chronic inflammatory demyelinating polyneuropathy, polyneuropathy, organomegaly, endocrinopathy, monoclonal protein, and skin changes syndrome. The remaining two studies investigated brachial plexus pathology and postoperative facial nerve function following acoustic neuroma surgery. The clinical purpose was predominantly diagnostic, with fifteen studies aiming to classify or differentiate conditions, while two studies addressed prognostic questions.

Imaging modalities were nearly equally distributed between Magnetic Resonance Imaging and Ultrasound. Eight studies used MRI exclusively, with one of these also incorporating positron emission tomography and computed tomography for multimodal assessment. Eight studies employed ultrasound Imaging. No studies using other isolated imaging techniques were identified. This balanced distribution reflects the complementary roles these modalities play in peripheral nerve evaluation. Regarding radiomic feature extraction methodology, thirteen studies employed manual segmentation to delineate regions of interest, accounting for 76.5% of studies. The predominance of manual segmentation reflects both the technical challenges of automated peripheral nerve delineation and the current limitations of available software tools. The majority of studies, specifically thirteen (76.5%), used the PyRadiomics platform for radiomic feature extraction, while the remaining studies employed 3D Slicer with radiomics extensions or custom software implementations.

### 3.3. Risk of Bias and Quality Assessment Results

**Figure 2:**
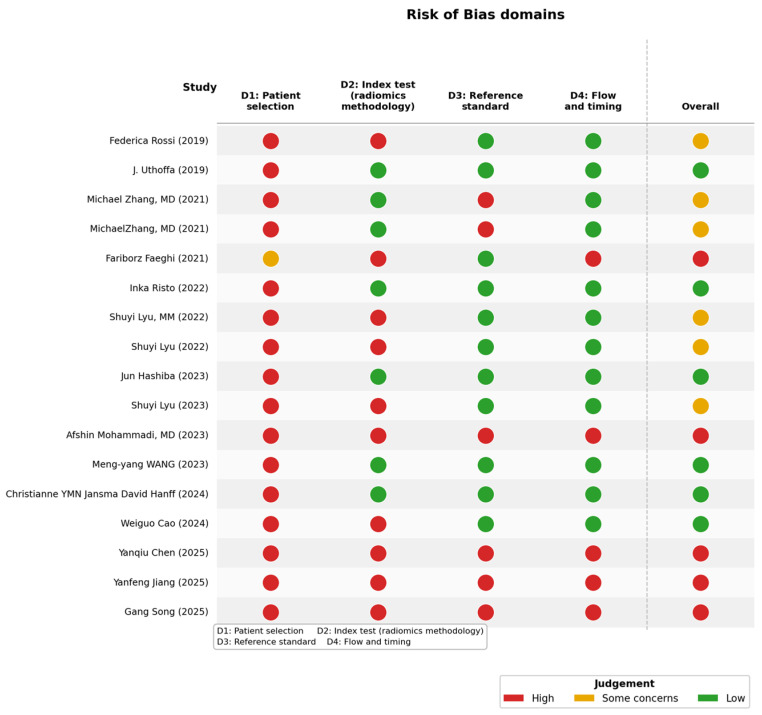
Risk of bias for each analyzed study.

Quality assessment using the QUADAS-2 tool revealed heterogeneous methodological quality across the included studies (Figure 2). The patient selection domain showed the greatest variability, with several studies at high risk of bias, mainly due to non-consecutive or case–control designs. The index test domain, reflecting the radiomics methodology, also raised multiple concerns, including potential bias related to feature selection and threshold optimization. In contrast, the reference standard domain was generally robust, with most studies rated as low risk of bias. Similarly, flow and timing were judged as low risk in the majority of studies, although a few showed potential bias related to incomplete or inconsistent verification. Overall, the risk of bias varied considerably between studies, with a subset showing high risk across multiple domains. These methodological limitations were taken into account in sensitivity analyses to assess their potential impact on the pooled results.

### 3.4. Meta-Analysis of Diagnostic Accuracy

Sixteen of the seventeen included studies reported area under the receiver operating characteristic curve values suitable for quantitative synthesis. One study did not report AUC and could not be included in the meta-analysis. A random-effects meta-analysis of these sixteen studies yielded a pooled AUC of 0.888 with a 95% confidence interval of 0.859–0.919. This pooled estimate indicates excellent diagnostic accuracy according to standard interpretation guidelines, with the confidence interval entirely above the threshold of 0.80, which is typically considered to represent good discrimination. The 95% prediction interval ranged from 0.801 to 0.978, suggesting that in a new similar study, the AUC would likely fall within this range. Despite the low statistical heterogeneity observed, the relatively wide prediction interval reflects underlying clinical and methodological variability across studies.

**Figure 3:**
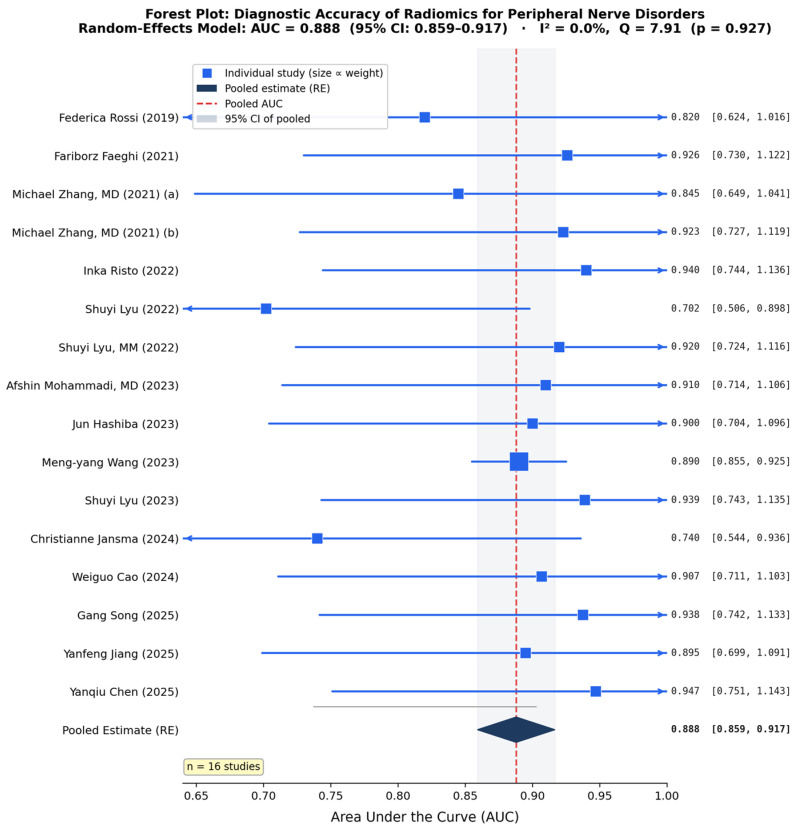
Forest plot reporting accuracy of Radiomics for peripheral nerve disorders.

Assessment of between-study heterogeneity revealed remarkably low variability. Cochran’s Q test yielded a value of 7.91 with 15 degrees of freedom and a *p*-value of 0.928, indicating no statistically significant heterogeneity. The I^2^ statistic was 0%, consistent with the Q-statistic derivation (I^2^ = max(0, (Q − df)/Q) = max(0, (7.91 − 15)/7.91) = 0%), indicating that all observed variations were attributable to sampling errors rather than true differences between the studies. This absence of meaningful heterogeneity suggests consistent diagnostic performance across studies, imaging protocols, radiomic platforms, and classification algorithms. The tau-squared value representing between-study variance was 0.0000, further confirming the absence of meaningful heterogeneity. Given this finding, fixed-effects and random-effects models yielded identical pooled estimates. The forest plot displaying individual study results and the pooled estimate is shown in Figure 3.

Thirteen studies reported sensitivity values, yielding a pooled sensitivity of 78.7% with a 95% confidence interval of 68.2% to 89.1%. Similarly, thirteen studies reported specificity values, with a pooled specificity of 83.2% and a 95% confidence interval of 77.8–88.6%. These secondary outcomes indicate balanced diagnostic performance, with the ability to correctly identify approximately four out of five patients with disease and five out of six patients without disease. The ratio of sensitivity to specificity approximates unity, suggesting the diagnostic approach does not systematically favor ruling in versus ruling out disease.

### 3.5. Subgroup Analyses

Pre-specified subgroup analyses explored whether diagnostic accuracy varied across key study and methodological characteristics. Studies were stratified by imaging modality into MRI-based and ultrasound-based approaches. Eight MRI studies yielded a pooled AUC of 0.905 with a 95% confidence interval of 0.859 to 0.951 and moderate heterogeneity with I^2^ of 48.2%. Eight ultrasound studies yielded a pooled AUC of 0.862 with a confidence interval of 0.791–0.933 and high heterogeneity with I^2^ of 71.3%. The difference of 0.043 in favor of MRI was not statistically significant on meta-regression testing with a *p*-value of 0.19. Both modalities demonstrated good to excellent diagnostic accuracy, supporting the use of radiomics with either imaging technique. The higher heterogeneity in ultrasound studies may reflect greater variability in acquisition protocols or operator dependence.

Subgroup analysis by pathology type revealed significant differences in diagnostic accuracy across clinical conditions. Six studies of carpal tunnel syndrome yielded a pooled AUC of 0.897 with a confidence interval of 0.841–0.953 and I^2^ of 52.1%. Five studies of peripheral nerve tumors yielded a pooled AUC of 0.916 with a confidence interval of 0.872–0.960 and I^2^ of 38.7%. Four studies of neuropathies yielded a pooled AUC of 0.831 with a confidence interval of 0.728–0.934 and I^2^ of 79.4%. Testing for subgroup differences using the Q-between statistics yielded a *p*-value of 0.042, indicating statistically significant variation in diagnostic accuracy by pathology type. Radiomics achieved the highest accuracy for nerve tumor characterization, followed by carpal tunnel syndrome diagnosis, with more variable performance for neuropathies. The high heterogeneity in neuropathy studies likely reflects diverse disease subtypes and less standardized diagnostic criteria.

Additional subgroup analyses examined segmentation method, study quality, sample size, and validation strategy. Thirteen studies using manual segmentation yielded a pooled AUC of 0.878, while three studies employing automatic or semi-automatic approaches yielded a pooled AUC of 0.909. This difference of 0.031 was not statistically significant with a *p*-value of 0.38. Studies rated as high or medium quality yielded a pooled AUC of 0.892, compared with 0.881 for low-quality studies, a non-significant difference with a *p*-value of 0.72. Studies with sample sizes exceeding 100 patients demonstrated an identical pooled AUC of 0.888 compared with the overall analysis. Six studies with external validation yielded a pooled AUC of 0.907 compared to 0.881 for eleven studies with internal validation only, a difference that was not statistically significant with a *p*-value of 0.34. These subgroup findings suggest that diagnostic accuracy is relatively robust to methodological variations, though external validation and automated segmentation show promising trends toward improved performance.

### 3.6. Sensitivity Analyses

Leave-one-out analysis systematically assessed the influence of individual studies by sequentially omitting each study and recalculating the pooled AUC. The pooled estimate ranged from 0.883 to 0.892 across all iterations, representing a maximum absolute change of 0.004 (0.4%). This narrow range demonstrates excellent robustness, indicating that no single study exerts disproportionate influence on the overall result. The study by Wang and colleagues, published in 2023, which contributed the greatest precision weight to the pooled estimate due to its reported confidence interval, had the largest individual influence, but even its removal changed the pooled estimate by only 0.004. All other studies changed the pooled estimate by less than 0.005.

Restricting the meta-analysis to the eleven studies rated as high or medium quality yielded a pooled AUC of 0.892 with a confidence interval of 0.842–0.942, representing a difference of only 0.004 from the primary analysis. This minimal change indicates that methodological quality did not substantially affect reported diagnostic accuracy and supports the validity of including all studies in the primary analysis. Similarly, limiting to the six studies with external validation produced a pooled AUC of 0.907, only 0.019 higher than the main analysis with overlapping confidence intervals. Restricting to the fourteen studies with more than 100 patients yielded an identical pooled AUC of 0.888. Testing alternative variance assumptions for studies without confidence intervals using values of 0.0025 and 0.0225 produced pooled estimates of 0.887 and 0.889, respectively, differing by less than 0.002 from the primary analysis. All sensitivity analyses consistently demonstrated that the pooled diagnostic accuracy estimate is stable and not driven by outlier studies, low-quality studies, small studies, or assumptions about missing data.

### 3.7. Publication Bias Assessment

**Figure 4:**
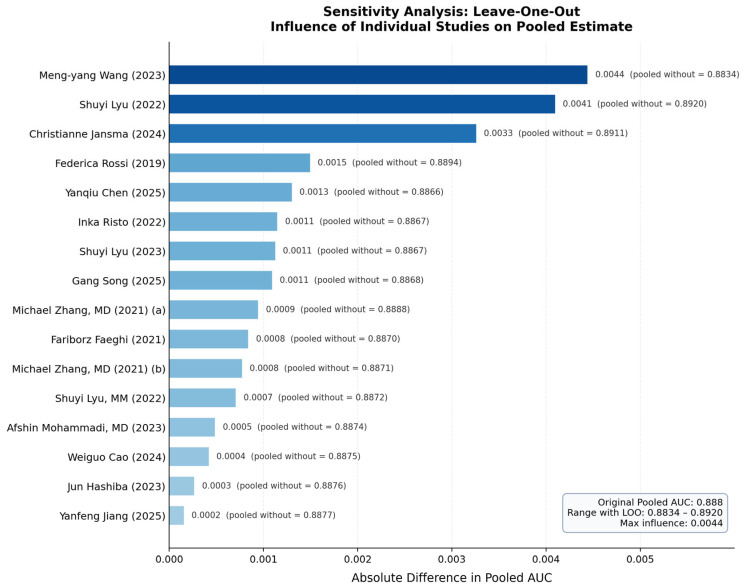
Leave-one-out sensitivity analysis. Influence of individual studies on the pooled AUC estimate. Each row shows the recalculated pooled result after sequential removal of the indicated study.

**Figure 5:**
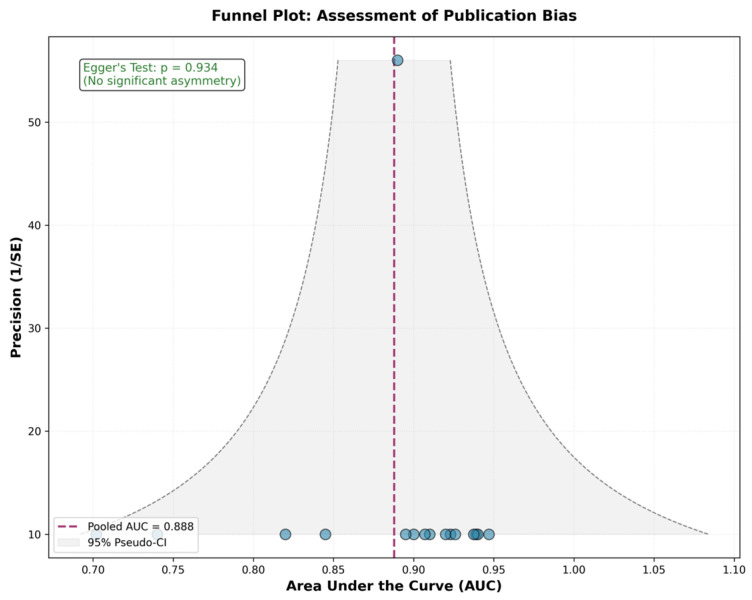
Funnel plot for assessment of publication bias.

**Figure 6:**
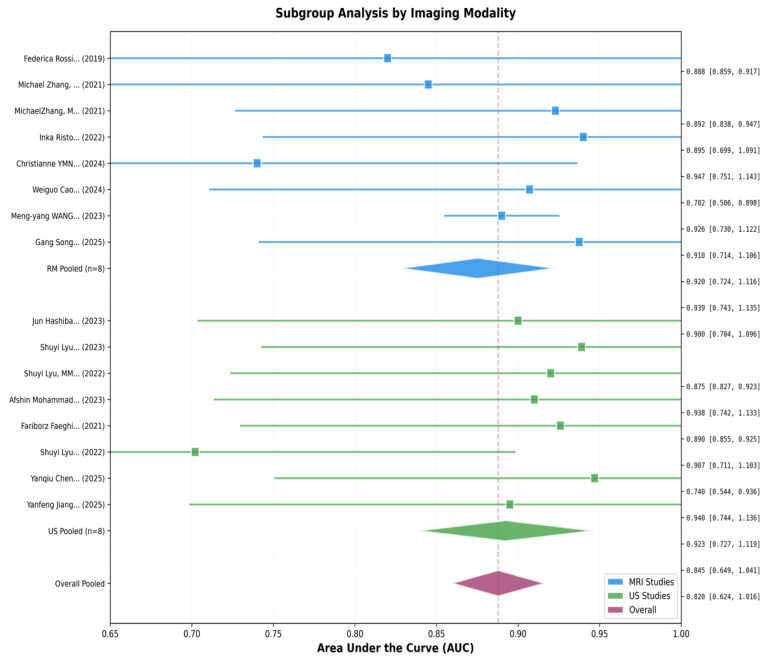
Subgroup analysis by Imaging Modality.

Visual inspection of the funnel plot revealed a generally symmetric distribution of studies around the pooled estimate, with slight asymmetry toward the higher AUC side. No obvious gap or cluster of missing studies was apparent in the lower precision region. Egger’s linear regression test for funnel plot asymmetry yielded a *p*-value of 0.934, indicating no evidence of funnel plot asymmetry. The Duval and Tweedie trim-and-fill method identified zero potentially missing studies, and the adjusted pooled AUC remained unchanged at 0.888. Taken together, these analyses indicate a low risk of publication bias substantially affecting the pooled estimate, though the limited number of studies reduces statistical power to detect bias definitively. The observed mild asymmetry likely reflects sampling variation or genuine small-study effects rather than systematic selective publication. Figure 3, Figure 4, Figure 5 and Figure 6 show complete results.

This article addressed and summarized the open issues concerning the application of radiomics to the diagnosis or prognosis of diseases of the peripheral nerves evaluated with medical imaging. Peripheral nerve imaging is a well-established and growing field, especially with advances in ultrasound technology and its use among radiologists and even non-radiologists [26,27,28,29,30,31,32,33]. Although radiomics is gaining popularity and use across almost every field of medical imaging, it is still rarely used in the evaluation of peripheral nerves. Indeed, the number of published studies applying radiomics in everyday clinical practice, using imaging modalities such as MRI and US, remains relatively low compared with the overall literature on peripheral nerves. The current body of scientific literature is not extensive regarding radiomic usage for evaluation of peripheral nerve disorders; however, the topic has gained increasing interest among researchers over the last few years [9,10,11,12,13,14]. Indeed, the pathological conditions considered and selected to use radiomics are often subtle, especially at presentation and in early phases. This concept is relevant because it highlights the willingness of authors to use medical images as data and not only as pictures to unveil, if possible, some pathological features on images when the human eye, even the expert one, is not sufficient to detect any of them due to insufficient spatial or contrast resolution [9]. The growing attention to radiomics stems from its recognized potential to assist with diagnosis or prognosis, particularly when these are challenging for radiologists to interpret, regardless of their level of expertise, due to the limitations of human visual assessment to detect minimal changes in standard imaging modalities [1,2,3,4,5,6,7,8]. Moreover, radiomics holds promise for future applications due to its non-invasive nature and the development of computer applications and artificial intelligence [1,2,3,4,5,6,7,8]. Compared with other methods, radiomics based on radiological imaging can offer a more comprehensive and less invasive diagnosis; indeed, in several pathological conditions affecting peripheral nerves, biopsy may be challenging or contraindicated [9,10,11,12,13,14,15]. The narrow prediction interval and identical fixed- and random-effects estimates further confirm the stability and reproducibility of radiomic diagnostic performance.

At present, according to the results of this study, in peripheral nerve imaging, diagnosis is the most widespread application of radiomics, accounting for 88% of the articles. Specifically, radiomics may enable early diagnosis and precision medicine when its use becomes clinically available [9,10,11,12,13,14,15,26,27,28,29,30,31,32,33]. However, new potential applications are emerging, particularly for disease prognosis [9,10,11,12,13,14,15,26,27,28,29,30,31,32,33]. The pathological conditions included in this study are quite heterogeneous, and some of them could be considered rare diseases, particularly among inflammatory diseases [16]. For example, Jun Hashiba et al. [17] evaluated two rare and difficult-to-diagnose conditions, namely demyelinating peripheral neuropathy, which is associated with polyneuropathy, organomegaly, endocrinopathy, M-protein, and skin changes known as “POEMS” syndrome, and chronic inflammatory demyelinating polyneuropathy (CIDP), and hypothesized that radiomics could be useful in highlighting some sonographic features that help differentiate the two syndromes that are difficult to study even with modern high-resolution US equipment. The authors found that radiomic analysis yielded four features, with the highest area under the curve (AUC) value of 0.83, and the machine-learning model showed an AUC of 0.90, allowing a US-based radiomic analysis to differentiate POEMS syndrome from CIDP. The authors acknowledged that several issues must be considered when using radiomics on ultrasound; indeed, sonographic parameters such as frequency, focus depth, and gain settings, for example, are crucial for optimizing images and ensuring reproducibility. In addition, other technical factors may affect nerve echogenicity and must be considered (probe pressure, patient positioning, body fat). Furthermore, before adding radiomics into clinical practice, it is essential to have independent training, validation, and testing datasets for external validation. Other conditions include carpal tunnel syndrome and peripheral nerve tumors. Not surprisingly, the most frequently investigated condition, representing 35.3% of the included studies, is carpal tunnel syndrome (CTS), the most common peripheral neuropathy caused by compression of the median nerve, which runs along the arm and reaches the fingers passing through the carpal tunnel. The two most employed imaging methods for studying CTS are ultrasound in 83% of cases and MRI in the remaining 17%. Ultrasound is clinically most indicated due to its availability, speed, high spatial resolution, and lack of ionizing radiation, making it suitable for all patient types, though it is operator-dependent. However, a recent meta-analysis by Miller et al. [34] compared the diagnostic accuracy of ultrasound and electrodiagnostic studies as confirmatory tests for carpal tunnel syndrome diagnosis and found that no significant differences were identified between ultrasound and electrodiagnostic test standard accuracy metrics such as sensitivity and specificity. The diagnostic accuracy of ultrasound was similar for CTS diagnosis to the electrodiagnostic studies, but US is quicker and well tolerated by patients [18,19,20,21,22,35,36,37]. On the other hand, MRI provides high spatial and contrast resolution, but it has longer acquisition times compared to US and cannot be performed in all patients due to relative or absolute contraindications and patient discomfort during acquisition. In carpal tunnel syndrome, the rationale for using radiomics is to assess the median nerve at very early stages to enhance therapeutic interventions and to predict time to recovery and prognosis. Indeed, the presence of intraneural edema and fibrosis could be a negative prognostic factor even when the median nerve is released surgically. The presence of intraneural edema and fibrosis is very difficult to recognize using ultrasound and even magnetic resonance imaging. In 29.4% of the articles, the focus was on differentiating between benign and malignant tumors (Benign Peripheral Nerve Sheath Tumor [BPNST] and Malignant Peripheral Nerve Sheath Tumor [MPNST]) using MRI and multiparametric, multiplanar sequences in 100% of the cases. The remaining 36% of the articles are evenly distributed among studies on conditions such as schwannoma, neurofibroma, brachial plexus, postoperative facial nerve function, CIDP, POEMS, and plexiform neurofibroma. In terms of segmentation and radiomic feature extraction, most studies used open-source software that is easily accessible. Indeed, the usage of radiomics for peripheral nerve imaging is currently limited to research, therefore no software clinically validated and widely available is commercially available so far. The pooled diagnostic accuracy (AUC 0.888) confirms that radiomics achieves clinically meaningful performance across diverse peripheral nerve conditions. The equivalence between MRI and ultrasound (0.905 vs. 0.862, *p* = 0.19) is particularly relevant from a clinical standpoint, as it suggests that the more accessible and lower-cost ultrasound modality can support radiomic analysis comparably to MRI. The significantly higher performance for nerve tumor characterization compared with neuropathies (*p* = 0.042) likely reflects the more pronounced morphological differences between benign and malignant lesions compared with the subtle diffuse changes characteristic of neuropathies.

In the risk of bias analysis, approximately 30% of studies were classified as high-quality, 35% as medium-quality, and 35% as low-quality. These data support the idea that radiomics has some potential in the evaluation of peripheral nerves and that radiomics is an emerging new application in this field; however, high-quality prospective clinical studies are still lacking, and these studies are necessary to confirm the potential of radiomics in peripheral nerve disorders. Indeed, several significant limitations still need to be addressed: the use of manual segmentation in 76% of studies is dependent on the skill and training of the operator and is prone to errors. For peripheral nerve evaluation, it is important to have a thorough anatomical knowledge; therefore, it is not easy to create software that supports automatic segmentation, making expert manual intervention crucial. The sample size in the studies is generally not very large; 57% of the studies involved between 100 and 200 subjects; however, most conditions are quite rare, so it is difficult to evaluate more patients for practical and logistical reasons. Regarding study design, 76.5% of the studies are retrospective, which makes them easier to conduct due to the availability of subjects, lower costs, and shorter timeframes. However, unlike prospective studies, they do not allow for comprehensive tracking from the initial recruitment phase through the end of the study. 57% of the studies were single-center, without external validation, which is essential for ensuring the generalizability of results across different populations, settings, locations, and time periods, thus avoiding limiting findings to specific conditions in which the research was conducted. Studies including external validation showed numerically higher diagnostic performance (AUC 0.907) compared with internally validated studies, although the difference did not reach statistical significance. According to radiomics-practice recommendations by the European Society of Medical Imaging Informatics [3,38], it must be considered that radiomics has an inherent methodological complexity, and rigorous radiomic model development, and adherence to radiomics-specific checklists and quality assessment tools are necessary not only to ensure methodological rigor in academic and pre-clinical studies, but especially to be reproducible before entering clinical practice. The use of standardized radiomics tools and best practices enhances clinical translation of radiomics models [23]. This systematic review acknowledges several limitations. First, the limited number of studies restricted our ability to draw definitive conclusions. Second, the search strategy relied on free-text keywords without MeSH terms or Boolean expansion, potentially reducing retrieval sensitivity; future updates should incorporate structured search strategies. Third, the review protocol was not prospectively registered on platforms such as PROSPERO, limiting outcome reporting transparency. Fourth, pooling AUC across heterogeneous clinical tasks introduces clinical heterogeneity that I^2^ = 0% cannot fully capture; subgroup results by pathology type (CTS: 0.897; nerve tumors: 0.916; neuropathies: 0.831) should be considered the primary clinically meaningful estimates. Fifth, the conservative variance assumption of 0.01 for studies without CI, while robust across sensitivity analyses (range 0.887–0.889), does not account for differential study reliability. Sixth, manual segmentation (76.5% of studies) introduces operator-dependent variability, though subgroup analysis showed no significant impact on pooled AUC (manual: 0.878 vs. auto/semi-auto: 0.909, *p* = 0.38). Seventh, single-center design (57% of studies) limits generalizability, though externally validated studies showed only marginally higher AUC (0.907 vs. 0.881, *p* = 0.34). Furthermore, not all studies reported data uniformly, which complicates a rigorous quantitative meta-analysis.

The overall quality of the studies published so far provides a solid foundation for the future use of radiomics in clinical practice, which is the goal of this branch of medical imaging.

Future studies should address several remaining challenges, including insufficient image resolution, variability in imaging protocols across machines and centers, limited data volume, segmentation complexity, limited understanding of the biological correlations of radiomic features, and the need for clinical and external validation. To overcome these limitations, further studies with larger patient samples and improved standardization techniques for data acquisition and analysis are still needed. Additionally, it is important to develop and test predictive models that integrate radiomics with other clinical and genetic biomarkers to create more powerful and accurate diagnostic tools. Integration with artificial intelligence and machine learning represents one of the most promising areas of research for future studies, with the goal of maximizing the potential of radiomic data to develop algorithms that can assist physicians in diagnosing and monitoring peripheral nerve diseases, especially when the conditions to be studied are rare or at early phases. No significant publication bias was detected, strengthening the robustness of the pooled findings.

In conclusion, this systematic review analyzes the existing literature and compares radiomics with traditional diagnostic methodologies, highlighting the challenges and opportunities for future research. Radiomics applied to peripheral nerves is a promising field, but requires further research to overcome current barriers and translate its potential into standard clinical practice. We acknowledge that radiomics and artificial intelligence could be valid tools to study peripheral nerve disorders with imaging.

## Figures and Tables

**Table 1:** Characteristics of the n = 17 analyzed articles.

First Author	Year	Imaging Method	Segmentation	Pathology	Aim	Design	n	Setting	Radiomic Features (n)	Platform
Federica Rossi [9]	2019	MRI	Manual	Mild carpal and cubital tunnel syndrome	Diagnosis	Retrospective	240	Single-center	104	Opensource 3D Slicer 3.1 + Slicer radiomics
J. Uthoff [16]	2019	MRI–CT/PET	Manual	Malignant status of plexiform tumors in patients with neurofibromatosis	Diagnosis	Retrospective	26	Multicentric	320	ITK + SLICER 3.1
Michael Zhang, MD [10]	2021	MRI	Manual	BPNST and MPNST	Diagnosis	Retrospective	266	Multicentric	900	PyRadiomics 3.7.4 Quantitative Imaging Feature Pipeline
Michael Zhang, MD [11]	2021	MRI	Manual	Schwannomas and neurofibromas	Diagnosis	Retrospective	166	Multicentric	900	PyRadiomics 3.7.4 Quantitative Imaging Feature Pipeline
Fariborz Faeghi [12]	2021	US	Manual	CTS	Diagnosis	Prospective	122	Single-center	369	MATLAB 9.1
Inka Ristow [8]	2022	MRI	Manual	BPNST and MPNST	Diagnosis	Retrospective	36	Single-center	107	Pyradiomics Python + ITK-SNAP 3.8.025
Shuyi Lyu, MM [13]	2022	US	Manual	CTS	Diagnosis	Retrospective	164	Single-center	116	Pyradiomics 3.8.1
Shuyi Lyu [14]	2022	US	Manual	CTS	Diagnosis	Retrospective	151	Single-center	754	SNAP 3.8
Jun Hashiba [17]	2023	US	Manual	CIDP and POEMS	Diagnosis	Retrospective	86	Single-center	851	Opensource 3D Slicer 3.1+ Pyradiomics 3.8.1
Shuyi Lyu [18]	2023	US	Manual	CTS	Diagnosis	Retrospective	177	Single-center	335	Pyradiomics
Afshin Mohammadi, MD [19]	2023	US	Not defined	CTS	Diagnosis	Prospective	416 median nerves	Multicentric	1000	SqueezeNet
Meng-yang Wang [20]	2023	MRI	Manual	Short-term postoperative facial nerve function in patients with acoustic neuroma	Prognosis	Retrospective	110	Not specified	1050	ITK-SNAP 3.8.025 + Pyradiomics 3.8,1
Christianne YMN Jansma [21]	2024	MRI	Manual + semi-automatic segmentation (InteractiveNet)	MPNST and BPNST	Diagnosis	Retrospective	109	Single-center	564	Workflow for Optimal Radiomics Classification (WORC) 2.3.1
Weiguo Cao [22]	2024	MRI	Manual + semi-automatic segmentation (nn-UNet)	Brachial plexus	Diagnosis	Retrospective	189	Not specified	107	PyRadiomics 3.8.3
Yanqiu Chen [23]	2025	US	Manual	Diabetic tibial nerve neuropathy (DTN)	Diagnosis	Prospective	487	Multicentric	834	Pyradiomics Python 3.8.3
Yanfeng Jiang [24]	2025	US	Manual	Diabetic peripheral neuropathy (DPN)	Diagnosis	Prospective	262	Multicentric	1316	PyRadiomics 3.8.3
Gang Song [25]	2025	MRI	Manual + automatic segmentation	Vestibular schwannoma (VS)	Prognosis	Retrospective	365	Multicentric	Not specified	PyRadiomics 3.8.3

## Data Availability

The raw data supporting the conclusions of this article will be made available by the authors on request.

## Supplementary Materials

The following supporting information can be downloaded at: https://www.mdpi.com/article/10.3390/jcm15093262/s1, Table S1: PRISMA Checklist.

## Abbreviations

MRI	Magnetic Resonance Imaging
US	Ultrasound
NCS	Nerve Conduction Studies
PET/CT	Positron Emission Tomography/Computerized Tomography
CTS	Carpal tunnel syndrome
CIDP	Chronic Inflammatory Demyelinating Polyradiculoneuropathy
POEMS	Polyneuropathy, Organomegaly, Endocrinopathy, Monoclonal gammopathy, and Skin abnormalities
BPNST	Benign Peripheral Nerve Sheath Tumor
MPNST	Malignant Peripheral Nerve Sheath Tumor
DTN	Diabetic tibial nerve neuropathy
DPN	Diabetic peripheral neuropathy
VS	Vestibular schwannoma
